# EEG Spectral Generators Involved in Motor Imagery: A swLORETA Study

**DOI:** 10.3389/fpsyg.2017.02133

**Published:** 2017-12-12

**Authors:** Ana-Maria Cebolla, Ernesto Palmero-Soler, Axelle Leroy, Guy Cheron

**Affiliations:** ^1^Laboratory of Neurophysiology and Movement Biomechanics, Neuroscience Institute, Université Libre de Bruxelles, Brussels, Belgium; ^2^Laboratory of Electrophysiology, Université de Mons, Mons, Belgium

**Keywords:** EEG, swLORETA, motor-imagery, time-frequency, ERSP, ITC

## Abstract

In order to characterize the neural generators of the brain oscillations related to motor imagery (MI), we investigated the cortical, subcortical, and cerebellar localizations of their respective electroencephalogram (EEG) spectral power and phase locking modulations. The MI task consisted in throwing a ball with the dominant upper limb while in a standing posture, within an ecological virtual reality (VR) environment (tennis court). The MI was triggered by the visual cues common to the control condition, during which the participant remained mentally passive. As previously developed, our paradigm considers the confounding problem that the reference condition allows two complementary analyses: one which uses the baseline before the occurrence of the visual cues in the MI and control resting conditions respectively; and the other which compares the analog periods between the MI and the control resting-state conditions. We demonstrate that MI activates specific, complex brain networks for the power and phase modulations of the EEG oscillations. An early (225 ms) delta phase-locking related to MI was generated in the thalamus and cerebellum and was followed (480 ms) by phase-locking in theta and alpha oscillations, generated in specific cortical areas and the cerebellum. Phase-locking preceded the power modulations (mainly alpha–beta ERD), whose cortical generators were situated in the frontal BA45, BA11, BA10, central BA6, lateral BA13, and posterior cortex BA2. Cerebellar-thalamic involvement through phase-locking is discussed as an underlying mechanism for recruiting at later stages the cortical areas involved in a cognitive role during MI.

## Introduction

Motor imagery (MI), or the mental simulation of specific actions (Jeannerod, 1994; Jeannerod and Decety, 1995), has increasingly been used as a research paradigm in cognitive neuroscience, both as a type of neurological therapy (Cramer et al., 2007; Grangeon et al., 2012), and as a technique in sports training (Wang et al., 2014; Di Rienzo et al., 2015). MI may be considered as a conscious mental operation which re-activates not only a motor scheme already stored in the brain but also a multi-sensory arousal related to the MI task (Grush, 2004; Ridderinkhof and Brass, 2015).

The study of MI itself encounters three major challenges: (1) the ecological environmental circumstances, (2) the complexity of the resting state dynamics, and (3) the confounding factors of motor inhibition. The dramatic development of brain computing interfaces (BCI) has enhanced the study of EEG patterns during MI, with the final intention of using brain signals to control artificial actuators (Cheron et al., 2012; Broccard et al., 2014; Zhang et al., 2016). The majority of these approaches were accomplished in a seated posture, which avoids any possible influence from postural control (e.g., during erect posture) and peripheral signals (multi-sensory input) that could make the MI closer to the real movement experience (Cebolla et al., 2015). Because of the complexity of the brain’s resting state dynamics (Wang et al., 2009; Ferrarini et al., 2011) out of which MI operation emerges (Zhang et al., 2015; Saiote et al., 2016), the choice of a control condition remains a challenge (Gusnard et al., 2001). Confronted with this complex situation, which involves a dynamic interplay between conscious and unconscious processes (Cheron et al., 2009; Jerath et al., 2015) an ecological virtual reality (VR) environment seems appropriate for the study of brain oscillations related to MI, as it was demonstrated that ecological visual scenes produced similar brain activation patterns in human individuals (Hasson and Malach, 2006). Furthermore, a VR environment allows one to control the timing of the visual trigger cues administrated to the participants, which may serve as a control of the MI effective realization. The presentation of an ecological scene may lead to the activation of additional areas, permitting a chronoarchitectonic mapping of the mental operation (Bartels and Zeki, 2004). MI task and the related real movement present both comparable patterns of the time-frequency domain measurements of ongoing oscillations, notably mu-rhythm event-related desynchronization (ERD) during motor planning, execution and MI and beta-rhythm ERD during voluntary execution and MI (Neuper et al., 2009; Yuan et al., 2010; Kilavik et al., 2014; Yi et al., 2016). As the spatiotemporal power values of these rhythms are differently modulated for the different limbs mentally moved or executed, the classification of the limbs is possible. This is widely used in the multiclass sensorimotor rhythm non-invasive BCIs approach (Coyle and Sosnik, 2015; Marchesotti et al., 2016; Zhang et al., 2016). It has also been shown that the spatiotemporal power distribution of theta, mu and beta oscillations used as input to a multiple linear regression based kinetic estimator or coupled to a feed-forward neural network gave better accuracy for motion trajectory prediction of hand motion BCIs than the potential time-series of the delta band (Korik et al., 2016a,b). Comparing MI to the related real movement highlights the confounding aspects of motor inhibition (Angelini et al., 2015). Taking into account such a constraint, we previously introduced a paradigm (Cebolla et al., 2015) in which MI was compared not to a real movement but to a control rest condition. Concretely, participants were asked to imagine holding a tennis ball in their dominant hand and throwing it as soon as a visual target appeared in a virtual tennis court. Then, while remaining in the same standing posture and with the control condition including the same visual information, the task was changed to simply remaining relaxed when the target appeared. We characterized two ERP components that were evoked by the visual cue and related to the MI: a first negativity peaking at 300 ms (N300) and a second one at 1000 ms (N1000). The N300 presented a more restricted central localization than the N1000, which expanded to the frontal and postcentral areas. Brain dynamics were approached by the event-related spectral perturbation (ERSP) and inter-trials coherency (ITC) measurements, which revealed that these ERP components were accompanied by a stronger ERD in the high-alpha/low-beta frequency bands and an event-related synchronization (ERS) in the theta band around the N1000 than during the control rest condition. In addition, three significant clusters of ITC appeared in the delta, theta and alpha during the first 500 ms of the MI. As the neural sources of the time-frequency signals cannot be directly inferred from the scalp localization of the electrodes analyzed (Makeig et al., 2004), we aim in the present study to estimate such time-frequency sources by applying a source reconstruction modeling (swLORETA, Palmero-Soler et al., 2007) on the specified temporo-frequency periods of interest that we have determined in our previous study at the scalp level. We hypothesize that the present MI paradigm will activate a complex network specifically responsible for the EEG rhythmic power and phase modulations which are oscillatory mechanisms of brain function that cannot be estimated from the extensive knowledge of fMRI anatomical involvement during MI (Beisteiner et al., 1995; Grafton et al., 1996; Decety, 1996; Gerardin et al., 2000; Dechent et al., 2004; Sauvage et al., 2013; Jiang et al., 2015). As the source modeling used here does not underestimate deep sources (Trujillo-Barreto et al., 2004), we expect it will similarly reveal the cortical, subcortical and cerebellar networks involved.

## Materials and Methods

### Participants and Conditions

The participants and conditions studied here make part of a previous work (Cebolla et al., 2015). They gave their written informed consent following the local ethics committee of the Université Libre de Bruxelles and conformed to the Declaration of Helsinki. Eleven (five females and six males, mean age: 20.5 ± 3.3 years) healthy, right-handed (96.3% ± 8.1 by means of the Handeness inventory (Oldfield, 1971) participated in the present study. They had previously been qualified as good imagers (5.95 ± 0.95 score) by means of the French version of the Movement Imagery Questionnaire-Revised (Loison et al., 2013). This instrument assesses visual and kinesthetic movement imagery ability and it comprises seven visual and seven kinesthetic items entailing the performance of movement, visual and kinesthetic imaging of that movement and then rating the ease or difficulty of generating that imagery on a 7-point scale from very difficult to see/feel to very easy to see/feel. Scores higher than five translates high imagery ability.

**FIGURE 1:**
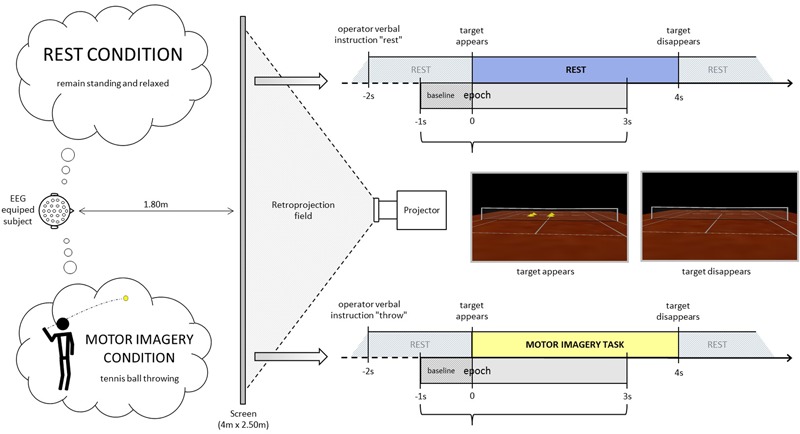
Experimental design. Rest (**up**) and motor imagery (MI) (**down**) conditions time-lines. Note that the visual information administrated with the VR scenario was the same in both conditions. In this way, the visual target (stimulus onset) in both conditions were the four vertical yellow cones appearing in the opposite left service court. Figure borrowed from Cebolla et al. (2015).

For the immersive projection, participants stood in front of a screen in a resting erected posture at a distance of 1.80 m from the center, where an empty tennis court was projected thanks to a rear projector BARCO RLM-W8 with a right orientation (3°), in order to perceive him/herself to be behind the right baseline of the court (**Figure 1**). The participants remained standing, at rest (baseline period). At least 2 s after the operator indicated “throw” or “rest”, an identical visual target (stimulus onset) appeared in the scenario (four vertical yellow cones), in the opposite left service court (with respect to the participant), for 4 s. Following the “throw” indication, the participants were asked to imagine holding a tennis ball in their dominant hand (right in the present population) and throwing it as soon as the target appeared, aiming at the target (four vertical yellow cones), in the opposite left service court. This task does not require any particular motor expertise. Participants were also instructed to stay focused on the imaginary ball they had just thrown until target disappeared. Following the “rest” indication, the participants were asked to remain relaxed and standing at rest when the visual target was presented in the virtual scenario. The visual information presented (four vertical yellow cones as visual target) was identical in both conditions (**Figure 1**, time-lime schemes). There were four or five series of 40 trials (20 per condition) randomly presented in a series. Breaks were planned between series. Participants did not mention feelings of tiredness during the recordings. If the operator observed noisier than usual online raw EEG signals, the participant was kindly asked to make a break and/or to stop the experiment. Thus 6 participants performed four series and five participants performed 5 series.

### Recording Parameters

The EEG was recorded with 128 channels (ANT neuro system) at a sampling frequency of 2048 Hz and with a resolution of 22 bits (71.5 μV per bit). An active-shield cap using 128 Ag/AgCl-sintered ring electrodes and shielded co-axial cables (5–10 electrode system placements) was comfortably adjusted to each participant’s head. All electrodes were referred to the right earlobe. In addition, three electro-oculograms (for horizontal and vertical EOG signals) and two electromyograms for detecting any activity related to real movement (anterior and posterior deltoids) were recorded.

### EEG Analysis

Off-line, data treatment and statistics were performed by means of EEGLAB software (Delorme and Makeig, 2004), ASA software (ANT neuro system) and in-house MATLAB-based tools. Initially, a 200 Hz low pass filter, a 512 Hz resampling and a 0.1 Hz high pass filter were applied. When necessary a 47.5–52.5 Hz notch was also applied. Then any artefactual portions of the EEG data were rejected by visual inspection. Synchronous or partially synchronous artefactual activity (mostly blinks) was detected and rejected by independent component analysis (ICA) on continuous data. Base line (-1 to 0) corrected epochs extracted from -1 to 3 s of the target apparition event were calculated. Visual inspection of the epochs allowed to reject those presenting extreme values. Finally, epochs presenting abnormal spectra (0.1–2Hz ± 50 dB and 20–40 Hz ± 5–100 dB) were discarded. After the artifact rejection process a total of 1788 epochs remained from the initial 1960 epochs (<10% rejected).

### Source Analysis

The method used here is described in detail in Cebolla et al. (2011).

#### swLORETA

Among the different sources reconstruction models, we have selected swLORETA (standardized weightened Low Resolution Brain Electromagnetic Tomography) because, as a distributed linear solution it does not assume a fixed number of sources and all the voxels in the search space are potential sources. swLORETA is a version sLORETA (Pascual-Marqui, 2002) which provides statistical parametric maps related to the reliability of the estimated current source density distribution. Compared to sLORETA, swLORETA enables the accurate reconstruction of surface and deep current sources even in the presence of noise and when two dipoles are simultaneously active. This is achieved by incorporating a singular value decomposition based lead field weighting that compensate for varying sensitivity of the sensors to current sources at different depths (Palmero-Soler et al., 2007).

The data were automatically re-referenced to the average reference as part of the LORETA analysis. A realistic boundary element model (BEM) was used for solving the forward problem (Zanow and Knösche, 2004). The solution was computed using 2030 voxels (5.00-mm grid spacing) and it was restricted to gray matter of cerebrum and cerebellum (based on the probabilistic brain tissue maps available from the Montreal Neurological Institute (Collins et al., 1994; Mazziotta et al., 1995). Talairach coordinates were obtained for every voxel by placing the corresponding Talairach markers onto the anatomical template (Lancaster et al., 2000). The final coordinates of the maxima values (*x,y,z*, Talairach coordinates) we provided for labeling the corresponding brain areas were based on the Talairach atlas. For the definition of cerebellar regions, we used the nomenclature of the MRI Atlas of the Human Cerebellum of Schmahmann (Schmahmann et al., 1999).

### Time-Frequency Sources

We calculate the brain areas that exhibit ERSP (Pfurtscheller and Neuper, 1994) and ITC (Tallon-Baudry et al., 1996) in the temporo-frequency periods of interest (**Table 1**). We used a classical baseline normalization or additive model for ERSP where the average baseline power calculated for each frequency band of interest is substracted from all the time windows at each frequency band of interest (Grandchamp and Delorme, 2011). The analytical signal was computed for each sensor channel for the *n*th trial of the experiment. After that the swLORETA was applied to the analytic signals for each individual trial. The ERSP and ITC in brain space over the n trials were then calculated as (Lin et al., 2004):

**Table 1:** Summary of the time-frequency periods of interest (Cebolla et al., 2015).

	ERSP		ITC		
𝜃	α	α/β	δ	𝜃	α
3–5 Hz 750–1150 ms	9–13 Hz 530–750 ms	9–17 Hz 1000–1350 ms	0.2–2 Hz 225–425 ms	4–7 Hz 480–520 ms	10–12 Hz 480–520 ms

*They are the same for the thee conditions: MI > baseline, rest > baseline and the MI > rest. The baseline period (-850 ms to -50 ms) preceded the stimulus onset.*

$$\begin{array}{c} \text{ERPS}(w,\text{​}t)=\frac{1}{n}\sum_{n\;=\;1}^{n}\text{diag}({\text{H}}_{\text{n}}(w,\text{​}t){\text{H}}_{n}^{*}(w,\text{​}t))\\ \text{ITC}(w,\text{​}t)=\frac{1}{n}\sum_{i\;=\;1}^{\text{n}}\frac{{\text{H}}_{\text{n}}(w,\text{​}t)}{\left|{\text{H}}_{\text{n}}(w,\text{​}t)\right|} \end{array}$$

where H_n_(w, t) is a row vector containing the analytic signal at time *t* and frequency *w* of the swLORETA estimates, *n* represents the number of trials, ERPS(w, t) represents the power spectrum of the swLORETA estimates, diag(M) is a vector formed by the diagonal elements of the matrix *M* and |*A*| indicate the norm of vector *A* and ITC represents the inter trials coherence of the swLORETA estimates.

Event-related spectral perturbation (Pfurtscheller and Neuper, 1994) measures variations in the power spectrum of ongoing rhythms at specific periods of time and frequency ranges. In ERSP, ERD (event related desynchronization) indicates a power spectrum reduction while ERS (event related synchronization) indicates a power spectrum increase. We also calculated the intertrial coherence (ITC) (Tallon-Baudry et al., 1996) because differently to ERSP, it measures consistency across trials of the EEG spectral phase at each frequency and latency window of ongoing rhythms. The time-frequency sources were calculated for the periods of interest previously reported (Cebolla et al., 2015) and summarized in **Table 1** for the thee conditions: MI > baseline, rest > baseline, and the MI > rest. The baseline period (-850 ms to -50 ms) preceded the stimulus onset.

**FIGURE 2:**
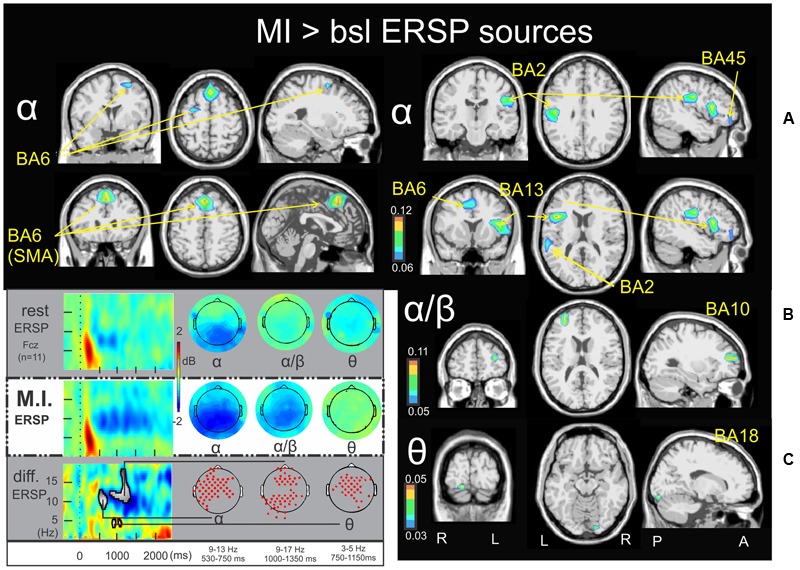
Motor imagery > bsl ERSP sources: Non-parametric statistical maps of the ERSP sources in the MI periods of interest with respect to the baseline. A recapitulative schema (borrowed from Cebolla et al., 2015) of such periods for a representative electrode (Fcz), in a two dimensions ERSP templates (ERS in red, ERD in blue) and the corresponding full scalp topographies are presented in the left inferior quarter part of the figures panel. Note that the condition concerned (MI) is highlighted with a white (instead of gray) background. In black background: (**A**; α) MI sources for the period of interest of 530–750 ms in the 9–13 Hz with respect to the baseline; (**B**; α/β) MI sources for the period of interest of 1000–1350 ms in the 9–17 Hz with respect to the baseline; (**C**; 𝜃) MI sources for the period of interest of 750–1150 ms in the 3–5 Hz with respect to the baseline.

**FIGURE 3:**
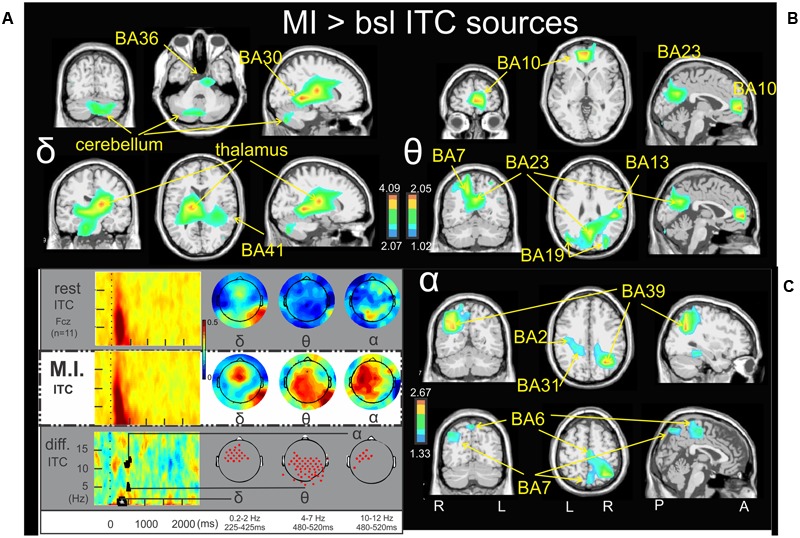
Motor imagery > bsl ITC sources: Non-parametric statistical maps of the ITC sources in the MI periods of interest with respect to the baseline. The related recapitulative schema (borrowed from Cebolla et al., 2015) for a representative electrode (Fcz), in a two dimensions ITC templates and the corresponding full scalp topographies are presented in the left inferior quarter part of the figures panel. Note that the condition concerned (MI) is highlighted with a white (instead of gray) background. In black background (**A**; δ) MI sources for the period of interest of 225–425 ms in the 0.2–2 Hz with respect to the baseline; (**B**; 𝜃) MI sources for the period of interest of 480–520 ms in the 4–7 Hz with respect to the baseline; (**C**; α) MI sources for the period of interest of 480–520 ms in the 10–12 Hz with respect to the baseline.

**FIGURE 4:**
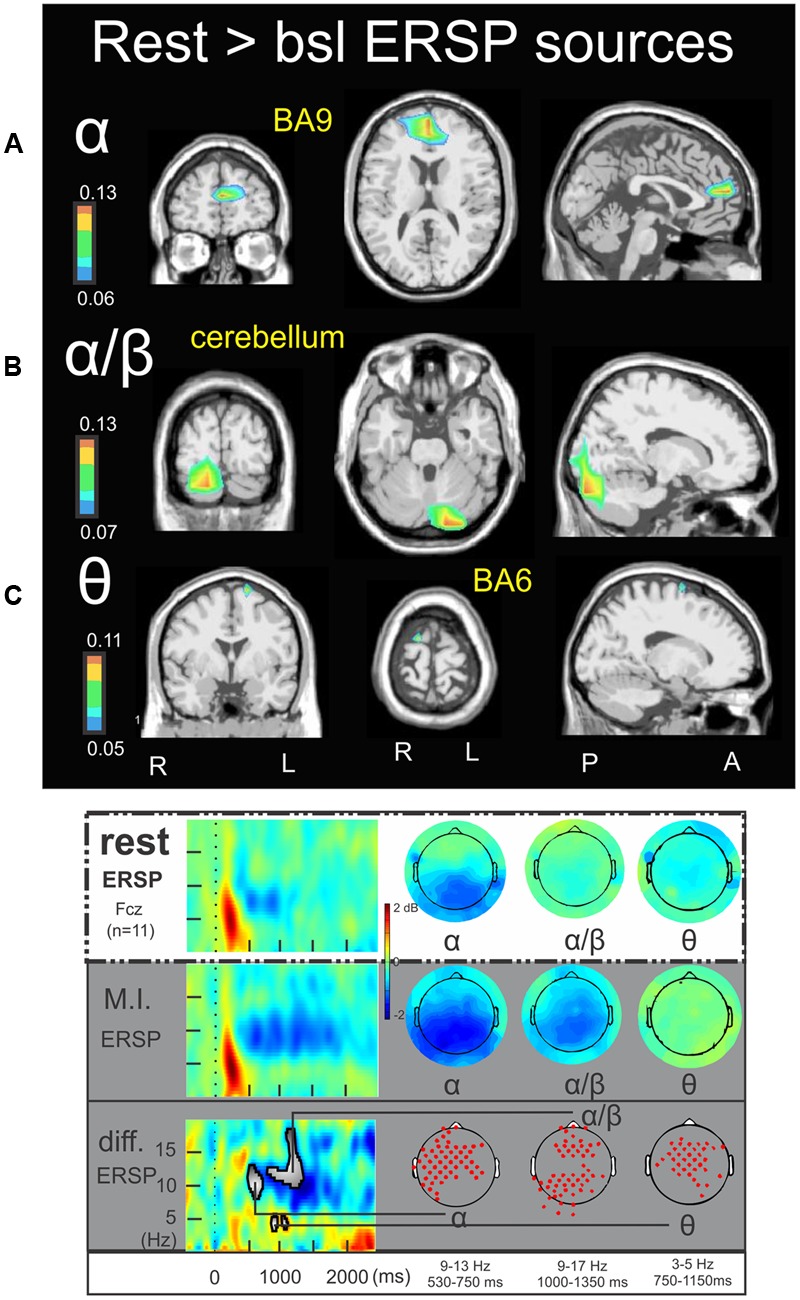
Rest > bsl ERSP sources. Non-parametric statistical maps of the ERSP sources in the rest condition with respect to the baseline. The related recapitulative schema (borrowed from Cebolla et al., 2015) for a representative electrode (Fcz), in a two dimensions ERSP templates (ERS in red, ERD in blue) and the corresponding full scalp topographies are presented in the inferior part of the figures panel. Note that the condition concerned (rest) is highlighted with a white (instead of gray) background. In black background: (**A**; α) rest sources for the period of interest of 530–750 ms in the 9–13 Hz with respect to the baseline; (**B**; α/β) rest sources for the period of interest of 1000–1350 ms in the 9–17 Hz with respect to the baseline; (**C**; 𝜃) rest sources for the period of interest of 750–1150 ms in the 3–5 Hz with respect to the baseline.

**FIGURE 5:**
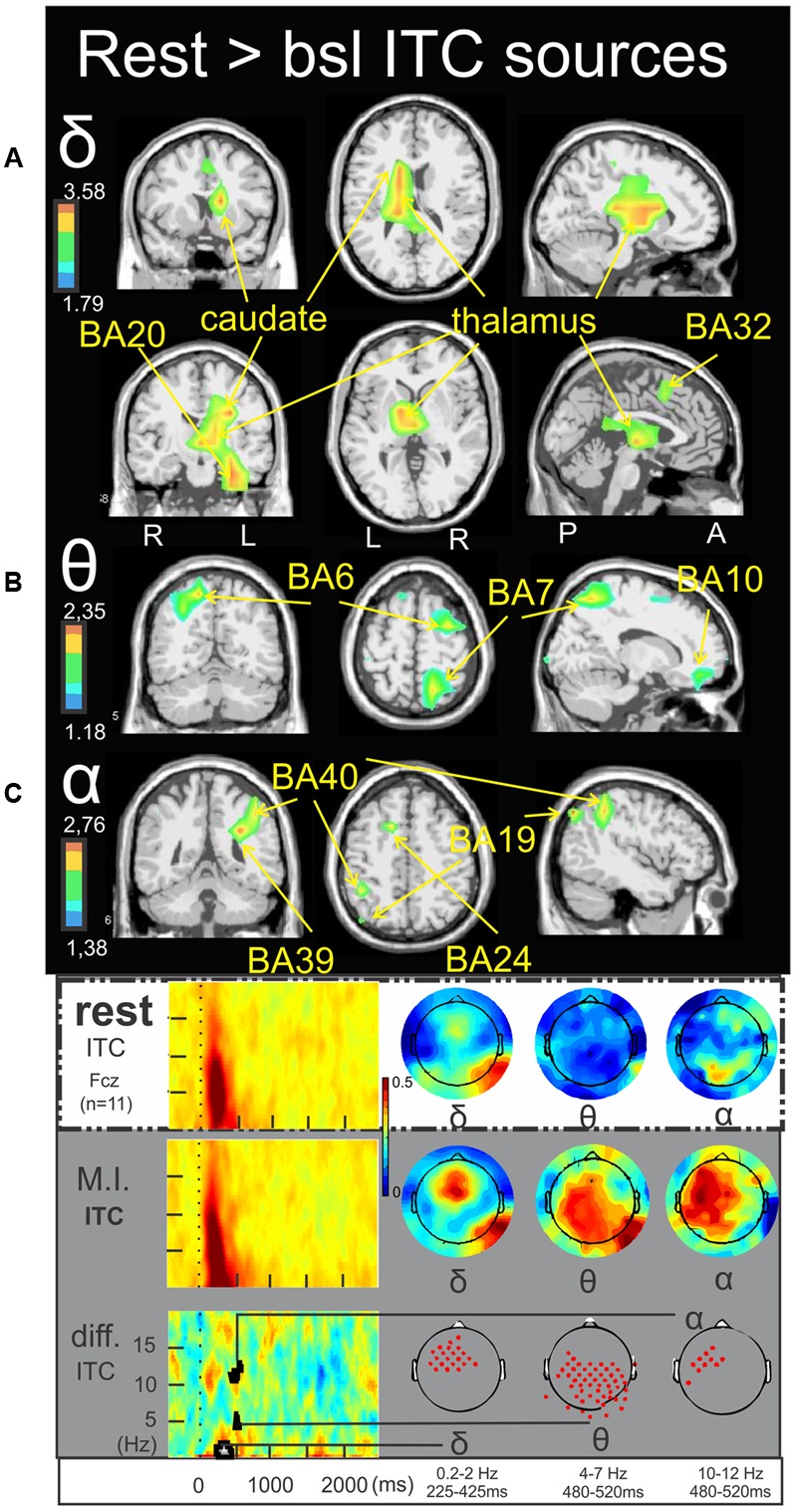
Rest > bsl ITC sources. Non-parametric statistical maps of the ITC sources in the rest condition with respect to the baseline. The related recapitulative schema (borrowed from Cebolla et al., 2015) for a representative electrode (Fcz), in a two dimensions ITC templates and the corresponding full scalp topographies are presented in the inferior part of the figures panel. Note that the condition concerned (rest) is highlighted with a white (instead of gray) background. In black background: (**A**; δ) MI sources for the period of interest of 225–425 ms in the 0.2–2 Hz with respect to the baseline; (**B**; 𝜃) MI sources for the period of interest of 480–520 ms in the 4–7 Hz with respect to the baseline; (**C**; α) MI sources for the period of interest of 480–520 ms in the 10–12 Hz with respect to the baseline.

In addition, to recapitulate these periods of interest, illustrations of the ERSP and ITC templates of a representative electrode FCz and full scalp topographies borrowed from our previous study (Cebolla et al., 2015) are reminded in **Figures 2–5, 7** where the concerned condition is highlighted in gray with respect to the other conditions in white background.

### Statistical Analysis

For the statistical analysis, we use the non-parametric permutation method (Nichols and Holmes, 2002) which is a statistical method that does not rely on the normality assumption and that controls for the false positives that may results from performing multiple hypothesis *t*-tests (one for each vowel). The permutation approach uses the data itself to generate the probability distribution for testing against the null hypothesis. We used paired *t*-test for swLORETA solutions to compare the conditions in the population, with a null hypothesis corresponding to the absence of difference between the compared conditions. We used the 95th percentile of the calculated permutation distribution for the maximal statistics, which defines the 0.05 level of corrected significance threshold. In other words, we can reject the null hypothesis for any voxel with *t*-values of the un-permuted T image greater than the 95th percentile of the permutation distribution of the maximal statistics (Holmes et al., 1996).

## Results

**Table 2** summarizes the results of the time-frequency sources obtained for the three conditions MI > baseline, rest > baseline and the MI > rest.

**Table 2:** Summary of the results of the time-frequency sources obtained for the three conditions MI > baseline, rest > baseline and the MI > rest.

	ERSP			ITC		
	𝜃	α	α/β	δ	𝜃	α
MI > bsl	BA18	BA2 BA6 BA13 BA45	BA10	BA30 BA 36 BA41 cerebellum thalamus	BA7 BA10 BA13 BA19 BA23	BA2 BA6 BA7 BA31 BA39
Rest > bsl	BA6	BA9	Cerebellum	BA20 BA32 caudate thalamus	BA6 BA7 BA10	BA19 BA24 BA39 BA40
MI > Rest	BA47	BA11	BA11	BA6 BA22 BA29 BA31 BA40 caudate thalamus cerebellum	Cerebellum	Cerebellum

### Time-Frequency Sources of the MI Condition >Baseline

**Figures 2, 3** illustrate the non-parametric statistical maps of the ERSP and ITC sources, respectively, plotted for all the participants in the MI condition periods of interest with respect to the baseline. A recapitulative schema (borrowed from Cebolla et al., 2015) of such periods for a representative electrode (Fcz), in a two dimensions ERSP (ERS in red, ERD in blue) and ITC templates and their corresponding full scalp topographies are presented in the left inferior quarter part of the figures panel. Note that the condition concerned (MI) is highlighted with a white (instead of gray) background. **Figure 2A** illustrates the significant ERSP sources (ERD in this case) of the alpha band (9–13 Hz) in the 530–750 ms period following the stimulus onset of the MI condition. Two significant clusters of ERD were found in the left cerebrum: in the middle frontal gyrus (BA6) (-26.6, -7.7, 55.1) and in the midline of the superior frontal gyrus (the supplementary motor area SMA, BA6) (-0.8, 18.3, 54.8). Other significant ERD clusters were found in the left inferior frontal gyrus (BA45) (-46.5, 35.3, 0.2), the left postcentral gyrus (BA2) (-45.3, -20.3, 27.6) and the left anterior insula (BA13) (-44.4, 6.7, 16.2). **Figure 2B** illustrates the significant ERD sources of the alpha–beta band (9–17 Hz) in the 1000-1350ms period of the MI condition, where one significant cluster was observed in the middle frontal gyrus (BA10) (-31.6, 44.6, 18.7). **Figure 2C** illustrates the significant ERSP sources (ERS in this case) of the theta band (3–5 Hz) in the 750–1150ms period of the MI condition, where one single cluster was found in the fusiform gyrus (BA18) (19.6, -86.7, -16.2). Concerning the ITC sources, **Figure 3A** illustrates those which account for the delta band (0.2–2 Hz) in the 225–425 ms period of the MI condition. Several significant clusters were found in the thalamus (-17.3, -16.2, 13.5), in the left parahippocampal gyrus (BA30) (-13.6, -37.7, -0.3), the uncus of the right limbic lobe (BA36) (16.0, -10.6, -28.9), the right superior temporal gyrus (BA41) (34.7, -30.4, 12.6), and in the cerebellum, with a cluster spreading from lobule VIIB (0.3, -69.1, -30.5) to lobule VI (-24.8, -69.7, -32.8). **Figure 3B** illustrates significant ITC sources in the theta band (4–7 Hz) during the 480–520 ms period of the MI condition, coming from multiple clusters situated in the left medial frontal gyrus (BA10) (-4.7, 49.8, 6.3), the right posterior insula (BA 13) (44.5, -28.5, 25.3), the right precuneus (BA7) (17.1, -61.4, 37), the right precuneus (BA23) (3.3, -60.1, 22) and bilaterally in the middle occipital gyrus (BA19) (-40.4, -76.9, 21,6 and 32.3, -76.4, 21.2 l). **Figure 3C** illustrates the significant sources in the alpha band (10–12 Hz) during the 480–520 ms of the MI condition: in the right paracentral lobule (BA6) (3.2, -23.5, 50.3), the left postcentral gyrus (BA2) (-41.2, -21.2, 29.3), the left limbic lobe (BA31) (-18.5, -39.0, 27.5), the right precuneus (BA7) (7.7, -72.8, 45.1), and the right middle temporal gyrus (BA39) (34.7, -58.4, 25.4).

### Time-Frequency Sources of the Rest > Baseline

**Figure 4** illustrates the significant ERSP cerebral and cerebellar sources in the alpha (**Figure 4A**), alpha–beta (**Figure 4B**), and theta (**Figure 4C**) bands during rest condition with respect to the baseline. The alpha ERD during the 530–750 ms period was characterized by a single cluster in the medial frontal gyrus (BA9) (-3.4, 43.5, 19.3) (**Figure 4A**). The alpha–beta ERS during the 1000–1350 ms period was characterized by a main source in the posterior lobe of the right cerebellum (Crus I) (16.5, -81.4, -26.0) (**Figure 4B**) and finally the theta ERD during the 750–1150 ms period was characterized by a single and discrete cluster in the left frontal lobe (BA6) (-15.3, -10.9, 69.0) (**Figure 4C**). Concerning the ITC sources, **Figure 5A** illustrates those which account for the delta band in the 225–425 ms period. Several clusters were found in the thalamus (-15.8, -5.8, 9.3), the caudate (-25.3, -17.0, 29.7), the anterior parahippocampal gyrus (BA20) (-32.8, -12.4, -22.5) and the left frontal lobe (BA32) (-1.9, 3.7, 47.1). **Figure 5B** illustrates the ITC sources in the theta band during the 480–520 ms period, with a cluster in the right middle frontal gyrus (BA6) (31.0, -7.0, 54.8), another cluster in the right precuneus BA7 (17.2, -62.8, 49.5) and third cluster in the left frontal lobe BA10 (-13.5, 39.7, -11.6). For the ITC sources in the alpha band during the 480–520 ms period, **Figure 5C** illustrates significant clusters in the left middle temporal gyrus BA39 (-30.2, -49.4, 25.8), the left inferior parietal lobe BA40 (-43.2, -49.0, 36.7) the left superior occipital gyrus BA19 (-42.3, -78.4, 34.1) and the anterior cingulate gyrus BA24 (-14.9, 1.9, 41.7).

### Time-Frequency Sources of the MI > Rest

**FIGURE 6:**
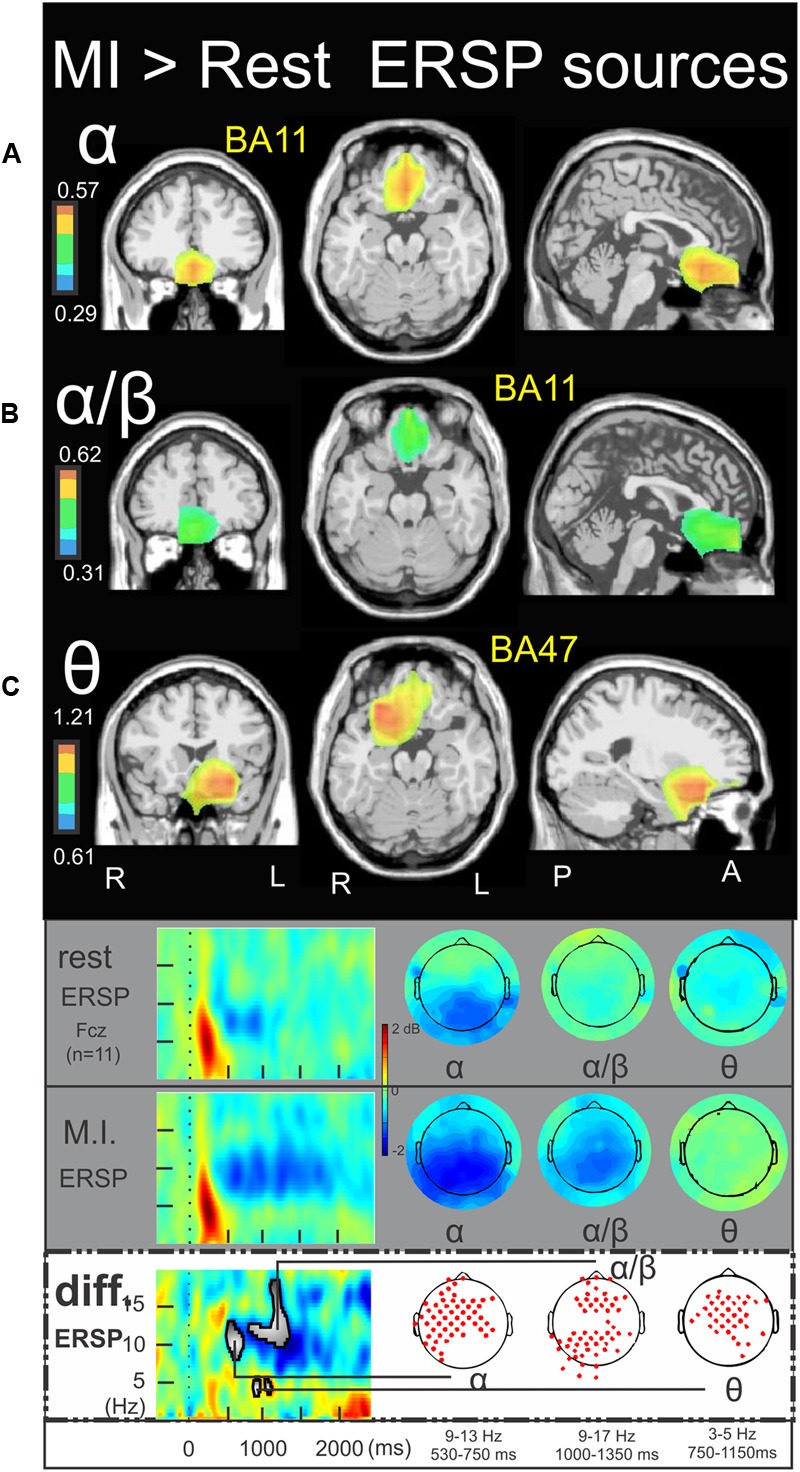
Motor imagery > Rest ERSP sources. Non-parametric statistical maps of the ERSP sources in the MI condition with respect to the rest condition. A related schema (adaptation from Cebolla et al., 2015) for a representative electrode (Fcz), in a two dimensions ERSP templates (ERS in red, ERD in blue) and the corresponding full scalp topographies are presented in the inferior part of the figures panel. Note that the condition concerned is highlighted with a white (instead of gray) background. In black background: (**A**; α) MI sources for the period of interest of 530–750 ms in the 9–13 Hz with respect to rest; (**B**; α/β) MI sources for the period of interest of 1000–1350 ms in the 9–17 Hz with respect to rest; (**C**; 𝜃) MI sources for the period of interest of 750–1150 ms in the 3–5 Hz with respect to rest.

**FIGURE 7:**
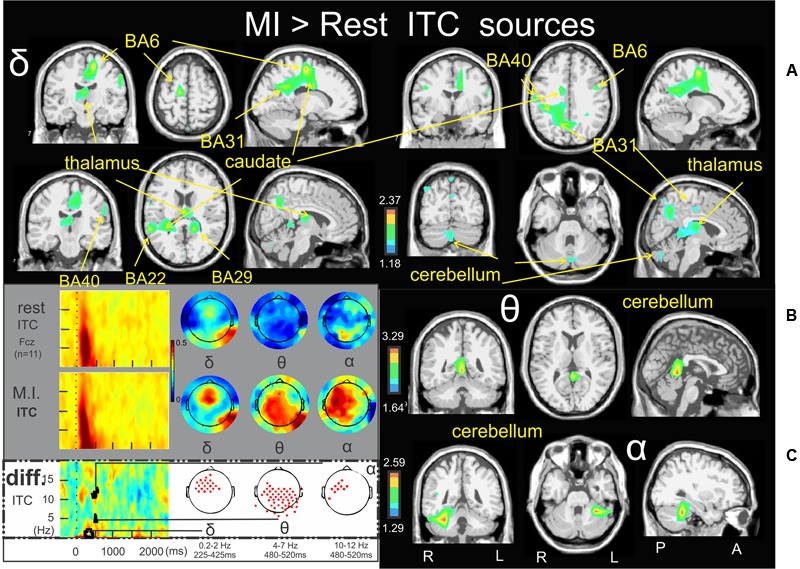
Motor imagery > Rest ITC sources. Non-parametric statistical maps of the ITC sources in the MI condition with respect to the rest condition. A related schema (adaptation from Cebolla et al., 2015) of such periods for a representative electrode (Fcz), in a two dimensions ITC templates and the corresponding full scalp topographies are presented in the left inferior quarter part of the figures panel. Note that the condition concerned (diff) is highlighted with a white (instead of gray) background. In black background: (**A**; δ) MI sources for the period of interest of 225–425 ms in the 0.2–2 Hz with respect to rest; (**B**; 𝜃) MI sources for the period of interest of 480–520 ms in the 4–7 Hz with respect to rest; (**C**: α) MI sources for the period of interest of 480–520 ms in the 10–12 Hz with respect to rest.

**Figure 6** illustrates the significant ERSP cerebral and cerebellar sources of the alpha (**Figure 6**), alpha–beta (**Figure 6B**), and theta (**Figure 6C**) bands during the MI condition with respect to the rest condition. In both the alpha and alpha–beta bands, a cluster of ERD was found in the left medial frontal gyrus (BA11) (-3.0, 27.1, -10.5, and -2.4, 39.2, -11.9, respectively). In the theta band, a cluster of ERS was found in the left inferior frontal gyrus (BA47) (-24.8, 16.5, -11.2). **Figure 7** illustrates the significant ITC sources of the delta (**Figure 7A**), theta (**Figure 7B**) and alpha (**Figure 7C**) with respect to the rest condition. These statistical maps revealed several significant clusters of delta ITC (**Figure 7A**): in the thalamus (3.8, -18.3, 13.1), the left caudate (-27.6, -36.9, 15.2), the left limbic lobe (BA31) (-13.0, 51.8, 24.6), the right precuneus of the occipital lobe (BA31) (1.0, -61.8, 25.7), the right limbic lobe (BA29) (16.5, -44.1, 10.6), the left superior temporal gyrus (BA22) (-56.6, -45.5, 11.6), the left inferior parietal lobe (BA40) (-58.3, -21.6, 29.4), the right precentral gyrus and the left medial frontal gyrus (BA6) (44.5, -3.9, 31.6, and -14.0, -18.7, 52.8, respectively) and the right cerebellum lobule VI (5.4, -72.4, -27.6). In contrast, a single source was identified for the theta ITC (**Figure 7B**) in the anterior part of the cerebellar vermis, lobule IV (1.6, -45.5, 0.9) and in the right anterior part of the cerebellum lobule V (31.8, -38.2, -24.4) for the alpha ITC.

## Discussion

The present study offers two complementary perspectives for characterizing the neural generators of the brain oscillations related to MI. In the first perspective, the MI and rest conditions were compared to their respective preceding baselines. In the second, the MI and rest states were compared independently of their baselines. We provide evidence that: (i) no matter which perspective used, the early phase locking (225 ms after visual cues) in delta oscillation related to MI was generated in the thalamus and the cerebellum; (ii) this delta wave was followed (480 ms) by concomitant phase locking in theta and alpha oscillations, generated in well-defined cortical areas and cerebellum depending of the perspective used; (iii) these phase-locked generators preceded the contribution of ERSP cortical generators (mainly alpha–beta ERD) from anterior in the frontal cortex BA45, BA11, BA10 to central BA6, lateral BA13 and posterior cortex BA2 related to MI.

Similarly to previous source modeling studies of MI (Rakusa et al., 2017), we first used the period preceding the MI task because this is a classical baseline, commonly used for the event related spectral perturbations in EEG dynamics studies (Makeig et al., 2004) and then we used a “rest” condition where the participants were asked not to move, and to remain relaxed and standing still while they were paying attention to the virtual scenario, because their body posture and the administrated visual targets were identical to the MI condition. We analyzed the impact of EEG oscillations on information processing by way of their response through the power and phase variations (ERSP and ITC time-frequency measurements (Makeig et al., 2004). In general, a power spectrum increase makes reference to “activation”, but in the case of alpha oscillation, power increase reflects “inhibition” and power decrease “release from inhibition” (Klimesch, 2012), for review). Independently of power, phase variations evaluate the discrete temporal characteristics of the EEG oscillations responses to stimuli. Phase coherence is considered as one of the main mechanisms of dynamic integration (Varela et al., 2001). Both local circuitry and long-range connections participate in the reentry mechanism in which cortical subsets of neurons can give rise to synchronized spiking activities that bind together the features present in sensory inputs (Edelman and Gally, 2013).

### Time–Frequency Sources of the Rest > Baseline

Initial delta (0.2–2 Hz) phase locking (in the 225–425 ms period) in the thalamus, the caudate and the left frontal lobe (BA32) may reflect the control process of maintaining an upright stance with cooperation through the theta and alpha phase-locking in the somatosensory association cortex (BA7, precuneus), in the temporal and parietal cortex (BA39, BA40) (Iacoboni et al., 1999), in the associative visual cortex (BA19) and later with the alpha–beta power ERS in the posterior lobe of cerebellum (Jahn et al., 2004; Visser and Bloem, 2005; Taubert et al., 2012). It is important to note that oscillations in the granule cell layer (4–25 Hz) of the cerebellum do not require movement in order to occur, as demonstrated in the immobile but attentive to the environment rhesus monkey (Courtemanche and Lamarre, 2005). The constant involvement of the prefrontal cortex accompanied by the activation of the anterior cingulate gyrus, superior and inferior parietal cortex, superior temporal gyrus, and thalamus may support the participant’s conscious visuospatial perception processes (Sturm et al., 2006; Libedinsky and Livingstone, 2011) related to the virtual scenario.

### Time–Frequency Sources of the MI > Baseline

#### Sources of the Phase Modulation

The early (225–425 ms) implication of the cerebellum and the (motor) thalamus (Prevosto and Sommer, 2013) through a delta phase locking mechanism may support the synchronizing role of the cerebellum through the thalamus to the cortical areas. Oscillatory coupling between the thalamus and cerebellum has been demonstrated by means of magnetoencephalography in patients suffering from essential tremor as a movement disorder (Schnitzler et al., 2009). Slow (1 Hz) oscillations have been reported in the cerebellar cortex of ataxic (Chen et al., 2009) and healthy anesthetized mice (Ros et al., 2009). Because the temporal opportunity window of synchronization is larger than in fast oscillations, slow oscillations allow for the synchronization of larger networks, since more distant neurons can be recruited (Whittingstall and Logothetis, 2009; Mazzoni et al., 2010). It is suggested that oscillations in the cerebellum serve not only for the synchronization of its internal activity but also to synchronize its activity to distal cerebral sensorimotor areas, as a large-scale network synchronizer (Llinás, 2011; Courtemanche et al., 2013; Cheron, 2016). In this line, cerebellar involvement by way of phase locking at low frequency might underlie the recruitment, via the thalamus, of the cortical areas expressing power decrease, such the SMA and the left frontal gyrus. Moreover, it has been shown by means of fMRI that the activity of this network comprising the cerebellum, the thalamus, the basal ganglia and the frontal gyrus increases during the MI of the standing posture (Jahn et al., 2004). This was implicitly part of the present task because participants remained standing during the MI of the simple task of throwing a tennis ball in the VR scenario of a tennis court. From another point of view, the involvement of cerebello-thalamic structures might reflect the cognitive control of behavior, as recent evidence shows that these structures have the requisite connectivity and activity to be able to mediate high level aspects of movement control (Prevosto and Sommer, 2013), for a review). The cognitive role of the cerebellum has already been suggested precisely in MI by using the regional blood flow technique to show the temporal organization of neuronal events related to cognition (Decety et al., 1990; Prevosto and Sommer, 2013). Following this cognitive perspective, the prefrontal cortex, which was activated here at a later period than the cerebellar-thalamic structures under the mechanism of an alpha power decrease during the MI condition, might reflect a top–down cognitive control of movement-related activity in thalamus, which constitutes an important aspect for volitional and selective gating of ascending inputs (Nadeau, 2008) which may, in turn, underlie the movement inhibition aspect of MI.

Involvement of the parahippocampal gyrus (BA30) and the perirhinal cortex (BA36) throughout the early delta ITC may support the participants’ visual exploration of the virtual scenario, as these areas have been, respectively, related to visual processes of high-demand discrimination and identification of the environmental stimuli (Murray et al., 2007). The subsequent visuo-spatial integration of the VR scenario’s characteristics and the participant may be supported by the theta phase-lock (480–520 ms) in the associative bilateral visual cortex (BA19), the right insula (BA13), the right precuneus (BA7, BA23), and the medial frontal gyrus (BA10). In this way, the precuneus elaborates information about egocentric and allocentric spatial relations for body movement control (Cavanna and Trimble, 2006). The precuneus is also involved in processing the integration of visuo-spatial information in MI tasks involving whole body movements in specific, challenging scenarios with obstacles (Ogiso et al., 2000; Hanakawa et al., 2003; Malouin et al., 2003).

At the same time (480–520 ms) an alpha (10–12 Hz) phase locking was localized in the right precuneus (BA7), the right middle temporal gyrus (BA39), the right paracentral lobule (BA6), the left postcentral gyrus (BA2), and in the left limbic lobe (BA31). The cortico-cortical projections from the precuneus to the lateral parietal areas and the premotor cortex play a role in the visual guidance of hand movements, hand-eye coordination and reaching (Caminiti et al., 1996, 1999; Ferraina et al., 1997). As there was not any movement production, the temporal and parietal areas implicated here could be related to proprioceptive information and to the coding of the precise kinesthetic aspects of the imaged movement as was proposed in the context of the neuron mirror system during observation and imitation movements (Iacoboni et al., 1999).

#### Sources of the Power Spectrum Modulation

The revealed sources accounting for the alpha ERD (530–750 ms) in MI corroborate previous general evidence from fMRI studies: in this way, the premotor cortex and SMA, well known in movement programming processes, have been robustly presented as the main actors of MI (Guillot et al., 2009; Jiang et al., 2015). The contralateral involvement of BA6 and BA2 also supports previous fMRI evidence that explicit MI of everyday hand actions is body specific with lateralization properties (Willems et al., 2009). It has been proposed that the activation of the parietal regions during tasks that combine both visual and MI (imaging upon objects) ensure the integration of somatosensory and visual processing (Sirigu and Duhamel, 2001). Even in the absence of visual and somatosensory input, MI induces contralateral superior parietal lobe activation in order to monitor the spatial position and orientation of the imagined limb (Wolbers et al., 2003). As previously described by fMRI in the MI of movement trajectories and of a moving target, the role of inferior frontal gyrus suggests a higher order forelimb movement control (Binkofski et al., 2000) which may be extrapolated here as participants were asked to focus on the imaginary ball they were throwing. The activation of the anterior insula also corroborates fMRI studies where MI was simultaneously coupled to action observation in a first-person perspective (Villiger et al., 2013), being associated with attentional and the control of goal-directed tasks as well as the awareness of causing an action (Nelson et al., 2010).

A later activation (1000–1350 ms) of the middle frontal gyrus (BA10) by the alpha–beta ERD was also revealed. It has been proposed that BA10 controls the subjects’ intentional and imitative motor behavior during cognitive demands through a functional cortico-muscular coupling at selective frequency bands (Babiloni et al., 2008). In this context, significant power spectral perturbations in the theta and gamma bands have been detected in BA10 accompanied by a low EEG-EMG spectral coherence as anticipated by the insignificant electromyography activity observed during a movement observation task (Babiloni et al., 2008). Similarly, one may presume a very low EEG-EMG spectral coherence during MI condition, which is characterized by the absence of real movement. In the same context, we speculated that BA10 activation in the alpha–beta rhythms might reflect the active control of movement inhibition in MI.

### Time–Frequency Sources of the MI > Rest

When the source location of the MI task was explored with respect to the rest condition, it was revealed that the phase locking mechanism in the delta oscillation initially implicated the thalamus, the basal ganglia (caudate), the premotor cortex (BA6), the inferior parietal lobe (BA40), the superior temporal gyrus (BA22) and the ipsilateral cerebellum (initially the posterior lobe and later the anterior lobe with theta and alpha phase locking). All these areas have been attributed to the MI in a first-person perspective, also referred to as kinesthetic MI, where participants experience realistic kinesthetic sensations (Amorim et al., 2000; Ridderinkhof and Brass, 2015). Besides corroborating the involvement of these areas, the present work supports the hypothesis of the delta phase locking mechanism (also the theta and the alpha phase locking in the cerebellum) acting as the functional mechanism underlying the MI. In kinesthetic MI, the left parietal cortex provides the kinesthetic aspects of the emulated action, which is subsequently mapped onto a motor representation in the premotor cortex which, in turn, communicates with the basal ganglia (caudate) for planning movement ideation. The cerebellum provides the kinematic details and precise timing of the motor emulation (Ridderinkhof and Brass, 2015). In our experiment, we did not explicitly ask the participants to focus on the kinesthetic consequences of the MI task over the visual aspects; however, using a first-person perspective may have entailed both kinesthetic and visual MI. Interestingly, the sources which characterize kinesthetic MI were revealed here when a comparison to the reference condition presented identical visual stimuli. Delta phase locking also implicated the limbic lobe (BA31, BA29). This may support a motivational or emotional aspect during the MI with respect to the rest condition. The limbic lobe is strongly connected to the frontal lobe (Rajmohan and Mohandas, 2007), which here was clearly affected by power variations (BA11, B47). This involvement might express a stand-by state between MI and the compared rest condition. Indeed, the frontal gyrus function enables the contingent interposition of two concurrent behavioral mental tasks (in this case the MI and the rest conditions) according to respective reward expectations (Koechlin and Hyafil, 2007).

### Limitations

Source reconstruction models have limitations by definition, as they use priors to ensure the uniqueness of the solution. Still source reconstruction from EEG signals is pertinent because differently to fMRI, EEG is a direct measure of the (global) real electrical brain activity. Following a classical approach, we have previously reported the time-frequency measures at the scalp level of MI (Cebolla et al., 2015). One important drawback of the scalp analysis derives from the reference choice. It has been demonstrated that a non-neutral reference influences the voltage waveform, the voltage weighted by the spatial coordinates of all the channels spectral distribution, the coherence and the network analysis (Qin et al., 2010, 2017; Chella et al., 2016). To overcome these limitations, it has been proposed a reference electrode standardization technique (REST) that approximately transforms multichannel recordings with a scalp point into real EEG data using an infinity neutral reference (Yao, 2001). In REST procedure, an equivalent source distribution on the cortical surface and a three-concentric-sphere head model are used to compute the transfer matrix which is used to re-reference scalp potential to an infinity reference (Yao, 2001). It is argued that the procedure is limited by the low accuracy of the head model (Nunez, 2010). However, several studies have demonstrated the accurateness of the REST procedure (Chella et al., 2016; Yang et al., 2017). REST procedure offers a solution to the reference problem but it does not directly inform about the neural generators. Confronted to the pitfalls associated at the scalp level with mixing of multiple cortical processes by volume conduction (Makeig et al., 2004), making impossible to make an inference to assess the involved generators, the present study focused on source reconstruction. Among the distributed linear solutions, swLORETA enables the accurate reconstruction of surface and deep current sources by incorporating a singular value decomposition based lead field weighting that compensate for varying sensitivity of the sensors to current sources at different depths. swLORETA has initially been validated with MEG (Palmero-Soler et al., 2007) and later with EEG signals (Palmero-Soler, 2016). A more recent validation was presented by Jennekens et al. (2013) in a study that evaluated EEG source localization by swLORETA for monitoring newborns with hypoxic-ischemic encephalopathy, using a standard anatomic head model and MRI. Hypoxic ischemic areas visible on MRI, corresponded well with swLORETA current density distributions. The use of individual MRI when performing statistics in a population are problematic because statistics in a population require a common template where all the individual values are considered. This problem can be approached by the wrapping methods but the error due to the wrapping methods is very difficult to quantify. In contrast, when using a standard MRI template in the inverse solution, this type of error is considered in the regularization parameters. In general, sources reconstruction methods require high density recordings a realistic anatomical model, a reliable solution with the lowest localization error, a high signal to noise ratio obtained with the experimental conditions and the validation of the method (Attal et al., 2007; Attal and Schwartz, 2013; Song et al., 2015). A gravity center measure for quantifying the localization error of very deep sources tested in a patch like solution could be more relevant than the classical Euclidean distance between the maximum of the current distribution and the position of the simulated dipole (Trujillo-Barreto et al., 2004). It must be recognized that whatever the used model, the exact representation of the full biological (structural and functional) features remain challenging. A realistic anatomical and electrophysiological model of deep structures named deep brain activity (DBA) model has been proposed in a study evaluating the sensitivity of MEG and EEG to signals from basal ganglia and the hippocampus (Attal et al., 2007). In addition, across all sampling density and inverse methods, adding samples on the inferior surface improves the accuracy of source estimates at all depths (Song et al., 2015). Although it could be argued that the thalamus is not a contributing source of EEG as it is mainly composed of stellate cells without a preferential neural organization facilitating the current cancelation, weak thalamic modulations on ongoing brain activity have been detected using the DBA model (Attal and Schwartz, 2013). Accordingly, the anatomical organization of the thalamo-cortical radiation (Squarcina et al., 2012; Alvarez et al., 2015) might be contemplated as a well oriented dipole at the level of their axonal cone being susceptible to synchronize to the MI task as the swLORETA results suggest in the present study. Interestingly and in the context of the DBA model, it has also been shown that during simultaneous cortical and subcortical activations, sLORETA can still detect hippocampal activity although such detection might include the creation of ghost deeper sources (Attal and Schwartz, 2013). An important effort must be realized in the future to precisely model the peculiar organization of the cerebellum which represents the highest concentration of neurons of the brain (Herculano-Houzel, 2009). Appropriated integration of the cerebellar–cortical organization including repetitive Purkinje cell dipoles, acting as open fields will probably reinforce the present indications of a possible contribution of the cerebellum in EEG recordings. In this context, deep sources located in cerebellum have previously been reported through inverse solutions models (Reyes et al., 2005; Elshoff et al., 2013; Stancak and Fallon, 2013; Proverbio et al., 2014, 2017; Cebolla et al., 2016). All thing considered, source localization of the time-frequency characteristic of EEG combined to the extensive knowledge of fMRI studies may contribute to assess the mechanisms of brain function at the same time as mathematical and practical methodological improvements of inverse solution models are still in progress.

### Futures Perspectives

The present results may contribute to BCI purposes in decoding the neural signature of MI. It is commonly considered that time-series of the low delta band-pass filtered EEG potentials provides the best accuracy in the motion trajectory prediction based BCIs as it is hypothesized that the trajectory of the movement is coded in the theta band (Robinson et al., 2015). However, this has been recently questioned when instead of times-series, time-varying spatiotemporal power distribution of theta, mu, and beta EEG oscillations is considered, being in line with the extensive use of mu and beta bands for classification of limbs of the multiclass sensorimotor rhythms based BCIs (Korik et al., 2016a,b). We provide evidence that during MI, delta EEG oscillation exhibits a phase-locking while alpha exhibits both power and phase-locking and beta exhibits power time-frequency mechanism (Cebolla et al., 2015). In line with previous studies, our present results suggest that the distinction between both ERSP and ITC measures might be of interest for specifying the relevant brain sources of the MI in BCI (Djemal et al., 2016; Santamaria and James, 2016). While most BCI models deliver command signals by sampling activity from very restricted scalp areas, time-frequency source reconstruction models, especially those with a distributed linear solution where the full brain and cerebellum are contemplated, may constitute an asset to BCI as they merge the full brain volume to distinct functional mechanisms of information (Yazmir and Reiner, 2016). Although it could be argued that performing MI and MI during BCI activates different neural networks, it has been shown that they activate a common large and distributed cortical and subcortical network including motor, premotor, supplementary and posterior parietal cortex (Marchesotti et al., 2017). From another new perspective, recent studies suggest that the transcranial direct-current stimulation increases MI-BCI accuracy (Ang et al., 2015; Hong et al., 2017). In this way, it has been shown that tDCS applied on motor cortex and cerebellum ten minutes before right-hand MI enhanced the accuracy of a classifier trained with the related mu and beta spectral power (Angulo-Sherman et al., 2017). Interestingly, it has been demonstrated that tDCS can impact not only mu and beta ERD in MI-BCI (Wei et al., 2013), but also the neural coupling and phase synchronization between the primary motor cortex and the supplementary motor area in MI (He et al., 2014) which reinforces the importance of characterizing and differentiating the time-frequencies sources of MI for BCI approaches. In addition to these specificities of EEG measurements, subjective and behavioral measurements as behavioral questionnaires and mental chronometry can be used as predictors of BCI control and its performance (Marchesotti et al., 2016).

## Conclusion

Through a reconstruction modeling of the EEG signals (swLORETA), the present study reveals the cortical, subcortical and cerebellar localization of the time-frequency characteristics (rhythmic power spectrum and phase locking modulations) of a MI of throwing a ball with the dominant upper limb in an standing posture, performed in an ecological VR environment. It was shown that, independent of the control condition (preceding baseline or a resting state in the same standing posture), an early delta phase locking was generated in the thalamus and cerebellum. This was followed by concomitant phase locking in theta and alpha oscillations generated in well-defined cortical areas and the cerebellum, depending on the control perspective used. Phase-locked generators preceded the contribution of cortical generators responsible for power modulations (mainly alpha–beta ERD) from the anterior frontal cortex (BA45, BA11, BA10 to central BA6, lateral BA13) and the posterior cortex (BA2) related to MI. Cerebellar-thalamic involvement through phase locking is discussed as an underlying mechanism for recruiting at later stages the cortical areas involved in a cognitive role during MI.

## Author Contributions

A-MC, AL, and GC analyzed the results. E-PS developed the swLORETA method, the related software packages and the statistical in-house statistic tools. All the authors contributed to the interpretation of the results. A-MC wrote the first draft of the manuscript and together with GC wrote the final manuscript.

## Conflict of Interest Statement

The authors declare that the research was conducted in the absence of any commercial or financial relationships that could be construed as a potential conflict of interest.
